# Draft Genome Sequence of *Lactobacillus plantarum* Strain SNU.Lp177 from Pig Feces in South Korea

**DOI:** 10.1128/genomeA.01184-15

**Published:** 2015-10-08

**Authors:** Eun Bae Kim, Gwi-Deuk Jin, Jun-Yeong Lee, Yun-Jaie Choi

**Affiliations:** a Department of Animal Life Science, College of Animal Life Sciences, Kangwon National University, Chuncheon, Gangwon-do, Republic of Korea; b Division of Applied Animal Science, College of Animal Life Sciences, Kangwon National University, Chuncheon, Gangwon-do, Republic of Korea; c Department of Agricultural Biotechnology, Seoul National University, Seoul, Republic of Korea; d Research Institute for Agriculture and Life Science, Seoul National University, Seoul, Republic of Korea

## Abstract

Herein we report a draft genome sequence for *Lactobacillus plantarum* SNU.Lp177, which was isolated from animal gut pig feces in South Korea. The draft genome of *L. plantarum* SNU.Lp177 contains 3,204,772 bp with a G+C content of 44.98% in 101 contigs (*N*_50_ = 116,595 bp).

## GENOME ANNOUNCEMENT

*Lactobacillus* strains are Gram-positive and non-spore-forming bacteria, and *Lactobacillus plantarum* is a representative probiotic species that is frequently used for humans and livestock animals. As inferred by its own name, however, the species is more frequently investigated in plant fermentations such as pickle, wine, and kimchi (1–3) than in the animal gut function.

As we are interested in *L. plantarum* strains from animal origins, we isolated many *Lactobacillus* strains from pig feces in South Korea (4). Only a limited proportion of the isolates were *L. plantarum*, while most other isolates were *Lactobacillus salivarius*. One of the *L. plantarum* strains, SNU.Lp177, showed antimicrobial activity against pathogenic *Salmonella*, K88-positive *Escherichia coli*, and *Listeria monocytogenes* that frequently infect piglets in South Korea and reduce growth performance of piglets (5). Such antimicrobial activity is regarded as one of the probiotic properties in the livestock industry. Thus, we sequenced the genome of this strain with probiotic potential.

*L. plantarum* SNU.Lp177 cells were harvested from a single colony. Genomic DNA was extracted by using a G-spin for bacterial genomic DNA extraction kit (Intron Biotechnology, Seoul). The genomic DNA was further processed to obtain a genomic library including ~300-bp inserts by using a NEBNext Ultra DNA library prep kit for Illumina (NEB, Ipswich, MA). The library was sequenced for paired-end 100-bp sequencing (2 × 100 bp) by using Illumina HiSeq2500 at the National Instrumentation Center for Environmental Management (NICEM, South Korea). A total of 398 Mbp quality-filtered reads were used to assemble a draft genome by using a genome assembler, Ray 1.7 (6), with a k-mer size of 31 bp. The draft genome contains 101 contigs (≥500 bp; total length, 3,204,772 bp; *N*_50_ length, 116,595 bp; maximum contig length, 216,279 bp; G+C content, 44.98%). A total of 3,102 protein code sequences (CDS) and 74 tRNAs were predicted by an annotation server, Rapid Annotation Using Subsystems Technology (RAST) (7). Only 41% of genetic features were covered by Subsystems, which was used to annotate genes in RAST. Three genes were associated with bile hydrolysis, which is necessary to protect *Lactobacillus* cells from toxic bile in the gastrointestinal tract. This genome will be useful for better understanding of the physiology of *L. plantarum* strains from animal origins.

### Nucleotide sequence accession number.

This whole-genome shotgun project has been deposited at DDBJ/EMBL/GenBank under the accession number LGIM00000000. The version described in this paper is the first version.
